# Research on Traditional Medicine: What Has Been Done, the Difficulties, and Possible Solutions

**DOI:** 10.1155/2014/495635

**Published:** 2014-06-12

**Authors:** Shirley Telles, Shivangi Pathak, Nilkamal Singh, Acharya Balkrishna

**Affiliations:** Patanjali Research Foundation, Patanjali Yogpeeth, Haridwar 249405, Uttarakhand, India

## Abstract

Traditional medicine (TM) is being used more frequently all over the world. However most often these are choices made by the patient. Integrating TM into mainstream health care would require research to understand the efficacy, safety, and mechanism of action of TM systems. This paper describes research done on TM and difficulties encountered in researching TM, especially when an attempt is made to conform to the model for conventional medicine. The research articles were PubMed searched and categorized as experimental, quasiexperimental, reviews, descriptive, historical, interviews, case histories, and abstract not available. The last part of the report provides suggestions to make research on TM more acceptable and useful, with the ultimate goal of integrating TM into mainstream healthcare with sufficient knowledge about the efficacy, safety, and mechanism of action of TM systems.

## 1. Traditional Medicine: The Existing Knowledge and Research

According to the World Health Organization atlas (2002), “traditional medicine (TM)” refers to health practices, approaches, knowledge, and beliefs incorporating plant, animal, and mineral based medicines, spiritual therapies, and manual techniques applied individually or in combination to treat, diagnose, and prevent illnesses or maintain wellbeing. It is worth noting that the description of TM given by the WHO in 2002 may have altered in some respects since then.

TM can be considered to belong to three main categories [1]. These are (i) codified medical systems, (ii) folk medicine, and (iii) allied forms of health knowledge [1]. Codified medical systems include great traditions which have evolved over 3-4 millennia and include Ayurveda, Siddha, and Unani in the Indian subcontinent and traditional Chinese medicine and acupuncture in China. These medical traditions have a unique understanding of physiology, pathogenesis, pharmacology, and pharmaceuticals which are different from Western biomedicine [2]. Perhaps because of this systematic approach these medical systems have been professionalized within the last millennia. Folk medicine is those traditional knowledge systems which are more often orally transmitted, have been generated by communities over centuries, and use components of the ecosystem which are locally available and accessible [1]. Folk medicine has not been formalized and is diverse and adaptable based on changing contexts. There are several similarities in the folk/indigenous medicine of widely differing, geographically distinct, communities. Allied forms of health knowledge include techniques which are related to wellbeing though they are not purely medical systems, such as yoga, tai-chi, qi-gong, and different meditations and breathing techniques [1]. The WHO published a global atlas to compile information on TM globally, in terms of policy, regulations, financing, education, research, practice, and use [3]. This provides a regional overview of TM, whether the systems are codified medical systems, folk medicine, or allied forms of knowledge. The description includes the use of TM in the African region, the Americas, the South East Asian region, the Western Pacific region (including Japan and the Republic of Korea), the European region, Eastern Mediterranean region, and Australian region. Globally the interest in TM, specific to that region as well as of other geographic areas, has increased due to easy accessibility, flexibility, relatively low cost, low levels of technological input, and relatively low side effects (WHO, 2002). Hence there is a definite need to mainstream TM into public health care. According to the WHO some of the major policy challenges include safety, efficacy, quality, and rational use of TM. Various policy measures have been and are being applied to the use of TM, in order to increase its acceptability, safety, and efficacy [4].

According to the WHO, the quantity and quality of safety and efficacy data on TM are not sufficient to meet the criteria needed to support its use worldwide.

There is no paucity of research on TM. A search in February 2014 of the bibliographic database PubMed, leads to 73,704 responses to “TM” as the search words, and the number has increased since then. An attempt was made to determine the number of papers published for different systems of TM such as Aboriginal, African, Alaskan, Ayurveda, Bhutanese, Caribbean, Inca, Maori, Mexican, Native American, Naturopathy, Persian, Siddha, South American, Tibetan, and Unani. It must be emphasized that this sample does not include all TM systems but attempted to cover those used often in different geographical locations.

In the present paper, the 300 most recent publications were categorized as (i) experimental (which included randomized controlled trials/nonrandomized trials, and detailed analysis of the active ingredients in herbal medicines and epidemiological studies); (ii) quasiexperimental which included observational studies; (iii) descriptive reports which mentioned the principles underlying the method of treatment; (iv) historical descriptions which detail the origins of the system; and (v) case histories or narratives. This is an arbitrary description, but the results, given in Table 1, are intended to give an approximation of the amount of research in each system of TM and the type of research being conducted.

## 2. Difficulties in Research in Traditional Medicine (TM)

While there is an increase in the use of TM worldwide, research in this area is inadequate, with serious difficulties in accepting the studies conducted [5].

Some of the main reasons why the studies conducted are considered flawed and inadequate are small sample sizes, variable or inconsistent results, and inadequate research designs [5]. Other problems include insufficient statistical power (possibly related to small sample sizes), poor controls, inconsistency of descriptions of the treatment or product, and lack of comparisons with other treatments or with a placebo or with both.

Most TM interventions use complex treatment methods which include botanical medications; individualized diagnosis and treatment; an emphasis on maximizing the body's innate ability to heal itself and a “whole systems” approach, wherein the physical, mental, and spiritual attributes of a patient are emphasized, rather than a focus on the disease as in conventional medicine (CM) [5].

Another difficulty encountered when designing a research study on a traditional healing method is that there are often differences in the forms, approaches, and nature (duration and intensity) of treatment, making it difficult to describe any TM in a single sentence, which would be understood to mean the same method by all people, everywhere. In the absence of such standardization, research on TM requires detailed descriptions of the interventions.

The criteria for including and excluding persons in a randomized control trial, RCT, differ between CM and TM; for example, having chosen TM as a therapy could be a criterion for exclusion, to reduce bias. If these exclusions are not observed the value of the RCT would be lowered. Other difficulties encountered are in randomizing patients, selecting a suitable placebo, and/or alternate intervention, as well as in masking and blinding. Randomization is very often difficult as patients have strong beliefs for or against TM and hence most often patients select to receive TM as a modality of treatment or alternatively choose to reject it. Another difficulty is that many of the treatments are carried out in specialized residential setting, under the supervision of a person trained in TM [6]. Quite often the residential center is in quiet surroundings, which have their own healing effects [7]. If the comparison group receives conventional treatment in their homes it is questionable whether the comparison between the two has any meaning, as the very fact that TM is carried out in a different setting [8] and with the personal attention of a TM healer could have a positive impact on the way the person feels and influences their subjective reports [9] and even possibly the outcome of the disease.

Another problem encountered with TM is selecting a suitable placebo. To begin with, interaction between the healer and the patient, which is usual in TM, can have a placebo effect [10, 11]. Apart from this when the participant receives an intervention such as chiropractic, massage, or acupuncture a sham treatment or placebo would be difficult to devise. This is all the more difficult if the patient is actively involved in the intervention, as in the case of yoga, practiced as therapy. A single study did attempt to use a device to simulate yoga breathing, as breathing through the device resulted in inhalation and exhalation being in a ratio of 1 : 2 automatically [12]. The sham device was identical to the active device but did not change respiration and hence was the placebo. Breathing through the active device did have a favorable effect in mild bronchial asthmatics, reducing their responsivity to histamine [12]. However practitioners of yoga may well question whether yoga breathing involves a change in the inhalation to exhalation ratio alone. Most of these techniques require subtle mental changes as well [13]; hence attempting to find a placebo for a TM intervention may actually result in evaluating limited components of the intervention.

## 3. Future Directions for Research in Traditional Medicine (TM)

The sections which precede this have demonstrated convincingly that there is the necessity for a new way to plan and conduct research on TM. The reasons why different guidelines are required for TM are due to the differences between TM and CM which are mentioned in Table 2.

Future directions include (i) policy making and standardization, (ii) training of researchers in TM with a combination of conventional research methods and those relevant exclusively to TM, (iii) financing research in TM and guidelines for writing and reviewing research grant proposals, and (iv) planning and designing studies in TM.

- Policy making and standardization are perhaps the most difficult challenge in TM systems (even those described as codified medical systems) [1]. There are vast differences in the methods used for any intervention and also in the way the healers are trained. Some courses, for example, may emphasize the physical aspects of the healing system, whereas other courses conducted elsewhere may emphasize mental and spiritual aspects. To make it possible for TM to be integrated into mainstream medical care it is essential that there be an attempt to standardize the healing method and courses involved in training those who deliver it. This would require having policies and specific nodal agencies to control and provide guidelines for this to be done properly.
- Training of researchers in TM in conventional research methods and those relevant to TM is an essential step to increasing research in TM. It is important to realize that many persons trained in using TM have a deep and abiding belief in the system of healing. This fact and the fact that they may not be trained in conventional physiology and anatomy may make them less suitable to carry out unbiased research on TM. Hence an important step is to select motivated yet unbiased persons who preferably have a basic knowledge of human anatomy and physiology. Many researchers in CM are trained to practice CM. Similarly if motivated persons trained in TM receive training in conventional research methods with the adaptations needed for TM [14], these trained persons would be ideal to conduct research on TM.
- Obtaining funds for research in TM is another essential step. Just as the NC-CAM of the NIH has allocated separate funds for research in TM, this is true for other countries as well. For example the Department of AYUSH, Government of India, India, has separate funds allocated for research in Ayurveda, Yoga, Unani, Siddha, and Homeopathy (AYUSH). However the format for research proposals is often more suited to research in CM. Apart from this the reviewers often have a distinguished career in CM with a partial or peripheral interest in TM. Hence only those research projects which investigate TM using the standards and norms set for CM are considered worth financing. Many areas related to understanding the mechanism underlying the benefits of TM may be considered “unscientific” or “dubious” by conventional researchers, as they involve concepts such as the subtle energy (*prana* in Indian medicine and* chi* in Chinese medicine). Nonetheless these concepts are a part of TM and if they are disregarded on the basis of being scientifically unacceptable, the risk is that TM would not be understood in its entirety. Hence an effort should be made to review all research grant proposals on TM by a panel comprising of experts in research on CM, researchers in TM, and persons with an in-depth knowledge of TM, but who are not biased in their approach to investigating TM.
- Planning and designing a research study on TM are challenging and require a change in the way research in this area is viewed. When planning efficacy trials it is necessary to accept that randomization and finding the proper controls are difficulties peculiar to TM and not found in efficacy trials on CM. Hence instead of randomization and attempting to have placebo controlled trials research on TM has to take into account various issues. For example, (i) the patient selecting TM with a belief in it could have its own placebo effect, (ii) the complexity of the interventions in TM often does not allow for a placebo, and (iii) the basic difficulty of comparing a whole life style changes with the approach of specific prescribed medicines in CM. A possible and indeed probably the only way forward in efficacy trials of TM is to adopt a “whole systems approach” where the entire set of practices which make up a TM healing system are compared with the conventional treatment, without any attempt to consider different aspects of the treatment, separately. Research on the mechanisms underlying the effects of TM also requires a shift in the way of thinking, so as to include complex concepts not used in CM such as “subtle energy” and “psychological and even spiritual benefits.”

Other research in TM, particularly related to herbology and plants used in healing, already follows conventional methods. Additional studies are required to understand the safety of herbomineral compounds and determine whether they have a risk of heavy metal toxicity or not [15].

Hence this brief report has attempted to provide an idea of the research which has been done in TM, the difficulties in applying CM research guidelines to TM, and possible guidelines for future directions which could make research in the area of TM worldwide more authentic as well as more scientifically rigorous. The ultimate goal would be to arrive at standardized systems of TM which can be integrated into mainstream healthcare, after having sufficient research-based information about their efficacy, safety, and mechanisms of action.

## Figures and Tables

**Table 1:** Details of articles on TM found in the PubMed search mentioned in the paper.

Serial number	Name of some TM systems	Articles found in PubMed on February 2014		Category							
		Total articles	Relevant articles	EX	QS	RV	DS	DS/HS	IN	CH	NV
1	Aboriginal	136	135	57	2	14	52	7	0	3	0
2	African*	2661	216	131	18	16	18	7	10	6	10
3	Alaskan	9	4	0	0	0	3	1	0	0	0
4	Ayurveda*	3514	300	172	17	24	43	16	0	13	15
5	Bhutan	20	16	8	1	0	3	0	3	0	1
6	Caribbean	306	106	56	8	7	9	21	0	2	3
7	Inca	5	4	2	0	0	2	0	0	0	0
8	Maori	30	21	6	0	0	8	0	1	1	5
9	Mexican	376	249	154	24	14	17	8	16	5	11
10	Native American	536	198	22	13	6	82	9	9	5	52
11	Naturopathy	1002	461	23	24	6	42	5	6	8	44
12	Persian	69	66	16	0	12	1	35	0	1	1
13	Siddha	203	113	59	17	0	15	3	2	0	17
14	South American	281	205	29	14	6	62	8	0	3	83
15	Tibetan	530	237	118	0	12	86	1	0	1	9
16	Unani	303	189	99	8	9	20	7	0	2	44

Note: *where total articles exceeded 1500, the 300 most recent articles were categorized.

EX = experimental.

QS = quasiexperimental.

RV = review.

DS = descriptive.

HS = historical.

IN = interviews.

CH = case history.

NV = abstract not available.

**Table 2:** Differences between CM and TM.

Areas which differ	Conventional medicine (CM)	Traditional medicine (TM)
(1) Mode of treatment	Primarily through medicine or surgery with additional information about precautions and side effects.	Includes polyherbal and mineral preparations, surgery, and guidelines encompassing the whole lifestyle (diet, mental attitude, physical activity, and even spiritual beliefs).

(2) Standardization	Well standardized so that it can be comprehended all over the world.	TM remains unstandardized. There are differences within a healing method; hence detailed descriptions are essential.

(3) Training of the practitioners	A well-defined system has been developed in each country.	There are differences in training program with respect to their content and duration.

(4) Quality of medicines	The medicines undergo rigorous testing and have to meet predetermined standards for safety which are set in each country.	Some of the codified medical systems, such as Ayurveda, do undergo testing for quality control and component analysis. However this is not rigorous and also it is not uniform within a country.

(5) Involvement of the healer	The healer who would be a trained physician or surgeon would need to know the detailed medical history of the patient and other details relevant to the disease before deciding and completing a course of treatment.	A healer of TM most often has to be involved closely with the patient's case history including the physical, mental, and even spiritual aspects. Diagnosis also involves interacting with the patient as do the treatments, which require the healer to participate in the treatment.

(6) Involvement of the patient	The patient has to be cooperative in the diagnosis, treatment, and follow up. Most often this involves taking specified medicines at specified times.	The patient actively participates in TM healing systems during the diagnosis, treatment, and follow up. While some TM methods such as massage require passive cooperation of the patient, others, such as yoga practiced as therapy, require the patient's active participation.

(7) Safety	The safety of CM is based on rigorous drug trials which go through several levels, from trials on experimental animals to final trials after approval on human subjects.	A few systems such as Ayurveda and TCM have had rigorous trials. However most TM preparations are not scrutinized with rigor.

(8) Adverse effects	Adverse effects for all medicines and surgical procedures are reported and made available to the medical community globally.	Adverse effects of TM systems are often not systematically documental or reported. This is an area in which considerable work remains to be done so that TM systems can have adequate legitimacy and be used widely.

(9) Efficacy and dosage	CM has details of the efficacy of the medicines and surgical procedures. Also, the dosages have been worked out taking into account factors such as age, body weight, and liver and kidney functions.	TM systems often decide the type and quantum of treatment based on individual factors. In some cases trying to apply the CM model to TM may reduce the usefulness of the TM system. Nonetheless there has to be a definite description of the factors which could determine TM efficacy and dosage.

(10) Mechanisms of action	The mechanisms of action of many CM methods of treatment are known.	Many TM are effective in healing but little is known about their mechanism of action. Research in this area is often made difficult by the fact that TM systems include subtle concepts such as “spiritual wellbeing,” “energy medicine,” and others which are not described in conventional medicine.
